# Model for understanding consumer textural food choice

**DOI:** 10.1002/fsn3.205

**Published:** 2015-02-02

**Authors:** Melissa Jeltema, Jacqueline Beckley, Jennifer Vahalik

**Affiliations:** 1 The Understanding & Insight Group LLC15501 Genito Road, Midlothian, Virginia, 23112; 2 The Understanding & Insight Group LLC3 Rosewood Lane, Suite 103, Denville, New Jersey, 07834

**Keywords:** Acceptability, consumer, food properties, mouth behavior, texture

## Abstract

The current paradigm for developing products that will match the marketing messaging is flawed because the drivers of product choice and satisfaction based on texture are misunderstood. Qualitative research across 10 years has led to the thesis explored in this research that individuals have a preferred way to manipulate food in their mouths (i.e., mouth behavior) and that this behavior is a major driver of food choice, satisfaction, and the desire to repurchase. Texture, which is currently thought to be a major driver of product choice, is a secondary factor, and is important only in that it supports the primary driver—mouth behavior. A model for mouth behavior is proposed and the qualitative research supporting the identification of different mouth behaviors is presented. The development of a trademarked typing tool for characterizing mouth behavior is described along with quantitative substantiation of the tool's ability to group individuals by mouth behavior. The use of these four groups to understand textural preferences and the implications for a variety of areas including product design and weight management are explored.

## Introduction

As part of the product development process (PDP), products are developed based on a marketing concept. It has long been recognized that developing products that match the marketing concept is important if they are to move successfully through the product development process. This includes linking brand attributes to product attributes and understanding the connecting threads between the two (Moskowitz et al. 2012). The beginning phase of the PDP is typically owned by the marketing department. Concepts are developed, evaluated, and chosen to guide product creation. The product researcher is typically not involved in the creation of the concept but is charged with developing and delivering a product that matches the concept. To assist the product researcher, the concept statement outlines the messaging that will be used to promote the product. Without a clear understanding of the product-based attributes that might be needed and how they can be developed as part of the product, the product will generally not be successful (Bhuiyan 2011).

Currently there is a trend to include the textural properties of the product in the message, for example, crunchy, chewy, or creamy. Texture has recently been identified as a mega trend (Sloan 2013). Key words around the texture message include crunchy, chewy, rich, thick, melted, soft, and creamy. Sometimes these words are used to connote more quality or emotional aspects such as freshness or mood, but at other times to connote texture preferences.

However, with concepts that employ texture messaging, the underlying belief is that (1) these textural characteristics are of interest to most consumers: (2) these textural words are clearly understood by both marketers and product developers; and (3) products can be easily optimized using current product development and sensory tools.

Current product development and sensory practices often involve the use of descriptive texture panels (Lawless and Heymann 2010). These panels are trained to evaluate the textural attributes using reference standards that have been determined to represent the attribute (e.g., crunchy). So, these methods assume that the attributes are well defined, can be represented by available standards, and that the use of these scales and standards will deliver products that match the textural attributes described in the concept statement.

The research reported here suggests that all of these assumptions are flawed, predominately because there are four distinct groups of consumers with each group having distinctly different texture preferences.

## Conceptual Foundations for Texture

It is well known that texture is important to product liking and preferences. Liking of a textural attribute (e.g., thickness or firmness), like taste, follows a parabolic curve (an inverted “U”, Moskowitz 2007). Optimum liking is at a textural level that matches the top of the parabola and liking decreases as the textural attribute is increased or decreased. The optimum for any textural attribute differs by food type. Additionally, it is well known that texture can be a major reason for food rejection (Drewnowski 1997).

This textural knowledge was slow in coming relative to other attribute discoveries and lagged behind flavor research. Through her early work, Alina Szczesniak, an early driver of textural research, found that individuals have low texture awareness. They see texture as an integral part of the nature of food and, therefore, have a limited ability to verbalize or distinguish textural characteristics as they are assumed to be fixed by the food (Szczesniak and Kahn 1971). When describing food, texture is often not mentioned; however, it is one of the strongest drivers of food aversion (Scott and Downey 2007).

If texture is such a strong driver of food aversion, why is texture awareness so low? Engelen and de Wijk (2012) suggest that individuals bring to each food a certain texture expectation. If that expectation is met, then there is no need to focus on texture. It is when that expectation is not met that there then is a reason for food rejection.

Due to the perceived inability of individuals to adequately notice or describe their textural experiences and the complexity of their texture perceptions, a preponderance of research seems to have focused on describing and measuring textural attributes sensorially and then relating the textural attributes to liking. Much of the research to understand the textural drivers behind liking has used highly trained panels to describe texture and then statistically relating those textural characteristics to consumer liking, collected separately. The belief is that the descriptive panel better understands these attributes and can measure them accurately. Therefore, these tools are predominately used to optimize a product's textural attributes (Lawless and Heymann 2010). Validation is typically conducted using consumers. This research is used validate that the textural attributes are optimized and determine if any further optimization is needed. In many cases, when assessing consumer acceptance, texture is assessed in conjunction with sensory attributes for flavor (i.e., taste and aroma). This is because flavor and texture are both drivers of food acceptance and the perception of one of these factors can change the perception of the other factor (Pacikora et al. 2003; Engelen and de Wijk 2012).

While a significant amount of research has been done to measure texture and to measure which textural attributes may impact liking, none of this research has focused on understanding the drivers of textural rejection or preference. Without this understanding, product researchers rely on mathematical models to optimize product formulations, without ever having a true understanding of why products are succeeding or failing with consumers.

## Oral Processing

Separately, there is a growing body of research on the oral processes during mastication to understand their effects on sensory perception. Differences in food manipulation and mastication have been found to affect sensory sensations. This research has highlighted some important findings, showing that chewing behavior varies by individual, but is consistent within an individual (Lassauzay et al. 2000; Po et al. 2011).

Brown and Braxton (2000) identified four different groups of people based on their efficiency in reducing the size of almonds and chewing gum. This research also showed that individuals use different mechanisms for the oral breakdown of food so that at any point, different groups of individuals would experience the samples differently. In this research, Brown and Baxton suggested that individual differences in the ability to manipulate and manage the product in the mouth may be a key driver of liking and personal preferences. However, the only link found in their research was a correlation between chewing force and preference.

Engelen and van Doorn (in Engelen and de Wijk 2012) also found that there were large individual differences in eating styles. In their research using semisolid foods (i.e., custard and mayonnaise), they asked participants to describe or draw what they did with the food in their mouths. Based on these self-descriptions, they identified four styles which they named: simple, taster, manipulator, and tonguer. Differences occurred with how the individual used their tongue, palate, and teeth.

Supporting the possible relationship between chewing behavior and preference, de Wijk, et al. (2003) found that for a given product, individuals achieved the highest sensory perception when they manipulated the product in their preferred style. Further research has also shown that the perception of attributes changed over mastication time and were associated with specific oral movements, suggesting that individuals use oral movements to enhance specific sensations (de Wijk et al. 2006, 2008). It has been suggested (Engelen and van Doorn in Engelen and de Wijk 2012) that individuals use an oral processing style that is balanced in terms of efficiency and perception, and that the processing style is also dependent on the food (in particular, the perceived pleasantness of the food). Therefore, the research suggests that individuals may use the chewing behavior that best provides the flavors and flavor intensity they are seeking (chewing foods differently is expected to change the flavor intensity more than the flavor itself).

Additionally it has been shown that chewing behavior is modulated by personality (Rey et al. 2007). This suggests that there may be genetic component to chewing behavior.

## A New Paradigm for Understanding Texture

This article suggests that there is a definite relationship between chewing behavior and food liking and satisfaction. Introduced here is a new model for understanding the drivers of texture preference and how to use that model in optimizing products. (Part of this text and data can also be found in a book chapter by Jeltema et al. 2014). This research suggests that Brown and Braxton were correct in their belief that the ability to manage food in the mouth is a key driver of preference. The results show that individuals have a preferred way to manipulate food in their mouths (mouth behavior) and that this behavior determines the food textures they prefer. *Therefore, it is not texture which is the key driver of liking, but textures that fit with the preferred mouth behavior*. Further, these findings indicate that individuals fall into four mouth behavior groups which we have named—Crunchers, Chewers, Suckers, and Smooshers.

## Theoretical Development of the Model

The insight into the existence of different mouth behavior groups came through reflection on quantitative findings and qualitative product research. These areas of inquiry began in 2001 and continued through the remainder of that decade. Based on these insights, mouth behavior groups were hypothesized and quantitative research was conducted to quantify and validate these mouth behavior groups. In this section, how these insights were obtained will be explained and then followed up with quantitative validation.

### An example of an observation that led to the insight

A product that was meant to be held in the mouth for a long time was given to research participants to evaluate their interest. These individuals were generally not interested in the product because most of them did not want to suck on it for a long time and they were unsure what to do with it in their mouths. This begged the question of what people do want to do with products in their mouths. Further observations (over 100 h) showed that individuals used products differently in their mouths, and that the way they were interacting with the products seemed to drive product acceptability.

### Exploration of the observations

Further exploratory qualitative research was conducted to understand the apparent differences in the ways individuals interacted with food and snacks. Individuals were asked to respond to a variety of statements aimed at understanding how they preferred to manipulate food in their mouths. They were asked to sort the statements (physically presented on cards with one statement per card) into three groups: (1) This is exactly like me; (2) This is somewhat like me; and (3) This is not like me. Some of the statements used are shown below:

- I like to suck on hard candy until it fully dissolves
- I usually break up hard candy quickly and swallow it
- I prefer hard crunchy cookies to soft chewy cookies
- I prefer soft creamy candies to hard candies

Of interest during this exploration was the complete lack of awareness of these behaviors by the individuals themselves until prompted through the card-sort. From this, mouth behavior appears to be a fundamental behavior which is not conscious. It has been well documented that many basic behaviors are unconscious, including repressed feelings, automatic skills, subliminal perceptions, thoughts, habits, and automatic reactions (Westen 1999).

Based on more of these qualitative listening and observing studies conducted over several years (more than 250 h of observation and listening), it was hypothesized that there were four major mouth behavior groups. As previously described, these four groups are: (1) Crunchers, (2) Chewers, (3) Suckers, and (4) Smooshers. These groups fell into two major modes of mouth actions. Mode one, represented by Crunchers and Chewers, were those who liked to use their teeth to break down foods. Crunchers were more forceful in their bite and preferred foods that broke up (fractured) on biting. Chewers liked foods that could be chewed longer (the length of time varied—there seemed to be “short” Chewers and “long” Chewers) and did not fracture on biting. Mode two, represented by Suckers and Smooshers, preferred to manipulate food between the tongue and roof of the mouth. They differed primarily in the hardness of preferred foods. Suckers liked harder foods (like hard candies and items that they could hold in their mouths) that could be sucked on for a long time. Smooshers preferred soft foods, such as creamy candies (like the wrapped candy called Cow Tales^R^ (Goetze Candy Co., Baltimore, MD) or puddings that did not require much mouth activity but would spread throughout the mouth and could be held in the mouth for a long time.

It is important to note that just because a person fell into a preferred group, does not mean that he/she rejected other mouth behaviors. While a person may generally have been a Cruncher, and often chose foods that could be crunched, he/she may also have some foods that he/she preferred to smoosh. However, in general, foods that allowed the preferred mouth behavior brought a higher level of satisfaction than foods that did not facilitate that mouth behavior. While participants were aware of their choices, they were generally unaware of the reasons that these food choices bring a higher level of pleasure (i.e., there was an unexpressed or unrealized need behind their behavior).

**Figure 1:**
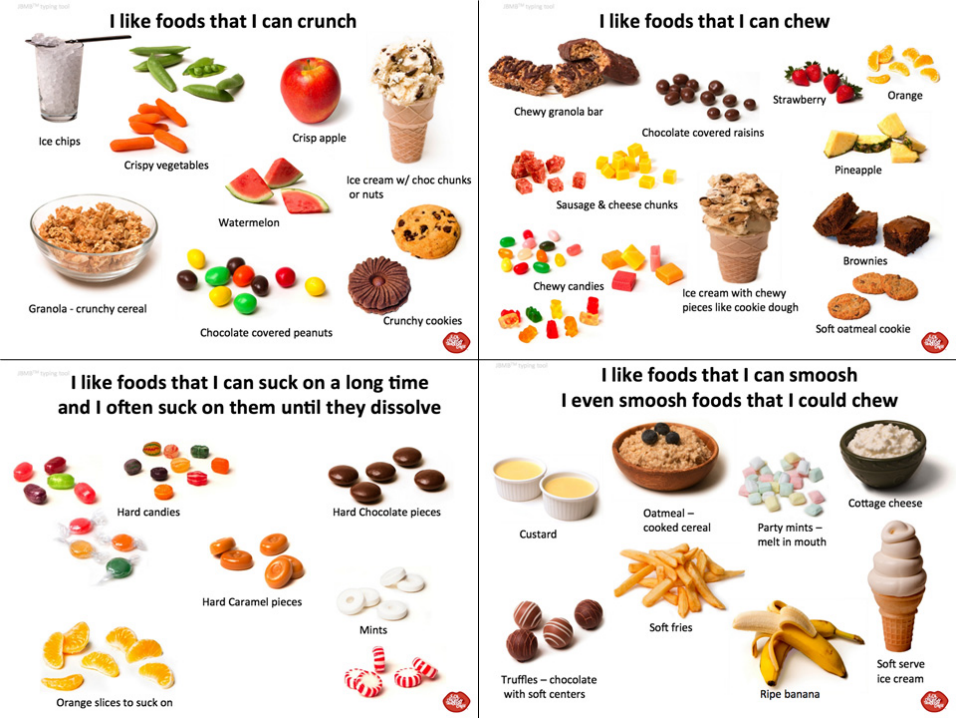
Graphic Mouth Behavior Tool (JBMB™)^a^. ^a^This figure is updated from that previously published in Jeltema et al. (2014).

The first qualitative confirmation came from many quite varied studies where a wide variety of products were available for people to choose. With all of these studies it was found that the particular product choice was clearly driven by the persons’ primary mouth behavior. While this finding was qualitative, there were numerous examples. One example of such research is shown below. In this particular qualitative research project, 20 individuals (participants at a dinner following an all-day meeting) were allowed to choose from several soups, snacks, salad toppings, and ice cream toppings from a dinner buffet. These foods were chosen to represent dinner options with varying textures. Participants were then typed for mouth behavior using the JBMB™ (Denville, NJ) tool—shown in Fig.1. Table1 indicates those choices which were most prevalent for a given group.

**Table 1:** Examples of products chosen by the different mouth behavior groups

Types of products chosen	Mouth behavior classification group
Chocolate with nuts, hard chocolate cookies with nuts, Cheetos^R^ and Ruffles^R^ (PepsiCo), raw broccoli	Crunchers
Gummy Bears, Starbursts^R^ (Wrigley Co.), Twix (Mars, Inc), Kettle and Cheetos^R^ Puffs (PepsiCo), soft granola bars	Chewers
Goat cheese, Buffalo mozzarella, French onion soup, whipped cream	Smooshers
Jolly Ranchers hard candies, Werthers Originals^R^ (August Storck KG) butterscotch pieces	Suckers

## Development of the Mouth Behavior Classification Tool

While it was found that the model was actionable (in that individuals could be consistently typed during qualitative interviews using the card-sort technique along with in-depth follow-up discussions on the reason for those choices), a tool that could more easily type people, yet still reproduce the assessments made during the in-depth interview, was needed. This proved to be difficult when done using a survey instrument, despite many efforts to construct such a survey. The reasons for this were many and included:

1. *Individuals are not aware of how they use food in their mouths*. Most people do not consciously think about how they interact with food, and therefore, often do not know what they actually do. “It would be easier if you gave me a food and then asked me what I was doing.” This unconscious awareness of such a primary need highlights the difficulties inherent in trying to understand behavior, emotion, and perception.
2. *Survey questions are often interpreted differently than was intended by the surveyor*. The individual may be thinking about a product grouping that is different than the surveyor intended. For example, one smoosher said “I only smooshed soft foods that can't be chewed”. She was thinking about meat, not something like a French fry.
3. *Individual behavior is not always consistent*. While a question such as “do you prefer crunchy hard cookies or soft chewy cookies” may generally work, a Cruncher may say, “Yes, most foods I like are crunchy, but with cookies I like a soft one that I can chew and smoosh.” Or they may say, “I choose a soft cookie because a hard one is dry and probably not as fresh.” Since surveys do not allow follow-up questions to resolve inconsistencies, accurately typing an individual through survey questions alone, was difficult.
4. *Individuals modify foods to meet their mouth behavior requirements*. When a food does not have a desirable texture, individuals will automatically modify their use of the product. For example, ice cream is much more likely to be eaten right out of the freezer by a Cruncher, while the other mouth behavior groups (particularly Smooshers) are more likely to let it soften before eating it. The same is found with cereals. While a Smoosher may eat a crunchy cereal, it is usually after it has been softened by the milk. On the other hand, individuals may modify the texture by adding other foods that will adjust the texture. For example, Chewers may add dried fruits like raisins and cranberries to a cereal or Crunchers, when eating a very soft cereal like oatmeal may add nuts and/or fruit. This modification of foods is particularly evident when an individual chooses to eat something less desirable (for reasons such as perceived health).

The reasons above illustrate how difficult it was to determine a person's mouth behavior from typical survey questions. Individuals had a low awareness of texture to begin with, and they were completely unaware of how they manipulated products in their mouths. Additionally, they would eat, for many reasons, products that were not optimal in texture, but would either modify their eating behavior or the product to make it more acceptable.

This observation on product modification was first made in the 1980's when work was being done by a breakfast food company on extruded cereals (J. Beckley 2013, Pers. comm.). The research participants would vary widely in their ready-to-eat cereal preparation. Some participants used only a very small amount of milk (to prevent the extruded cereal from becoming soggy) while others used a lot of milk and let the cereal get soft before eating it. This led to the creation of a methodology for “bowl-life testing”. Bowl-life testing was a form of over-time observational evaluation of the texture and flavor properties of the cereal. It was an early attempt to combine the evaluation of texture with other sensory properties to understand why a specific cereal might or might not be rejected by someone with a particular texture preference (crunchy, crispy, or soggy) and to establish metrics to measure the texture of a specific cereal over time when consumed with a specific type of milk, such as skimmed or whole milk.

Table2 illustrates the types of questions that were initially used in the last few years to type individuals for mouth behavior. The types of questions used and the reasons for inconsistencies are also shown in the table.

**Table 2:** Examples of questions used in the initial survey to place individuals into Mouth Behavior Groups^1^

Type of question	How well did question work in typing person?	Suggested reasons for inconsistencies
Ranking or choice of behavior—suck, smoosh, chew, crunch	Fairly well	Individuals are often not aware of what they do with foods in their mouths—it is subconscious
Chewy versus crunchy cookie	Not well	Crunchers often picked a chewy cookie because it was felt to be fresher and higher quality
Chew cookie completely versus chew and then smoosh	Fairly well	Some Chewers will smoosh certain foods, and a soft cookie is sometimes one of them
Soft creamy versus hard candy	Not well	Those who do not like food stuck in their teeth will not pick a soft creamy candy unless they are thinking of chocolate

1 This table was previously published in a book chapter (Jeltema et al. 2014).

The survey tool that was first developed was found to clearly identify approximately 75% of those sampled for mouth behavior type, with the other 25% being somewhat questionable as to which group they belonged.

## Refining the Tool

As a result of the findings from that survey tool, another approach was taken. Instead of basing results on simple differentiating statements, it was felt that an approach that indicated mastication patterns would be less susceptible to error. Therefore, a pictorial (image) solution was generated. Images have been found to be a good way of accessing the unconscious mind. “Rather than stating its meanings to us in specific logical terms, the unconscious mind portrays its messages to us in images and symbols” and “Dynamics within the unconscious can be known through imagery that may be visual, verbal, auditory…”(Progoff 1963).

Participants were shown four groupings of foods and asked which was “most like you.” This indicated their preferred mouth behavior because the foods shown were those that most easily allowed that behavior. The use of multiple foods removed the difficulty that arose with a particular food or flavor being disliked or handled differently in the mouth. Participants were also asked if any of the groups were “not like me at all” which would indicate a rejection of a given mouth behavior. This became the JBMB™ (Jeltema/Beckley Mouth Behavior) Classification Tool that is shown in Figure1.

## Validation of the Classification Tool

To validate this tool, a stepwise confirmatory procedure was undertaken. Twenty interviews were first conducted using the graphic tool. This was followed by the traditional survey tool for mouth behavior. Lastly, participants were interviewed to understand inconsistencies (those whose survey question answers were not consistent with any given mouth behavior or which were unexpected due to the pictorial mouth behavior chosen). This was done to determine their true mouth behavior, following the belief that these in-depth interviews provided the most accurate assessment of mouth behavior. In all 20 cases, the graphical solution was found to match the determination by interview and was deemed by the investigators to be that person's correct mouth behavior. This indicated that the pictorial determination of mouth behavior was much less susceptible to error than the survey.

To quantitatively validate the tool, an online survey was conducted in the United States among 500 males and females, ages 15–65. A prescreener was used to facilitate balance across age, gender, and region according to the US Census. The survey consisted of the JBMB™ Classification Tool along with a word-based survey tool that asked a variety of questions such as those shown in Table2. Responses were first compared across groups using chi-square analysis to determine whether the different mouth behavior groups were answering the questions differently. Examples of some of the survey responses that showed significant differences (*P* < 0.10) are shown in Table2. A discriminant analysis was then run using the JBMB™ mouth behavior classification as the Y variable and using the questions from the word survey as the *X* variables. This analysis showed that there were indeed different groups of individuals that could be separated using the data (*P* < 0.0001). The word survey questions were able to correctly classify individuals 75% of the time. The groups varied in their percentage of correct classifications. Chewers were correctly classified 82% of the time, while Smooshers were only correctly classified 67% of the time. The overall correct classification was in agreement with the qualitative findings that the word survey misclassified individuals approximately 25% of the time. It was not surprising that Smooshers were the most often misclassified by the word survey, as it had been found in the qualitative research that this group had the lowest awareness of how they manipulated food in their mouths. Therefore, based on these findings, the model appeared to be robust and the tool accurately able to type individuals.

## Results of Quantitative Study

### Proportion of mouth behavior groups

The proportion of each mouth behavior group from this survey (*N* = 500) indicates that the predominant groups were chewers and crunchers. Suckers were by far the smallest group (Table3).

**Table 3:** Mouth behavior classifications based on a survey (*N* = 500)^1^

Mouth behavior	Chewer	Cruncher	Sucker	Smoosher
Individuals classified in each group (%)	43	33	8	16
Individuals who reject each mouth behavior (%)	10	16	45	29

1 This table was previously published in a book chapter (Jeltema et al. 2014).

While it is important to know the type of mouth behavior most desired by any individual, it is equally important to know what mouth behaviors are rejected by individuals. Just as Suckers were the smallest group, it also turned out to be the behavior that was also most likely to be rejected by individuals (Table3).

As indicated earlier, the original example discussed for mouth behavior centered on evaluation of a product that was meant to simply be sucked. From the current survey data, it is clear that this product would not have a large following. Only 8% of the population prefers products that will be sucked on for a long time, while 45% reject products where the design limits them to this mouth behavior. This of course does not imply that only 8% of the population will eat a hard candy. Individuals who are not Suckers will simply choose to break it up a lot sooner.

### Reported behavior by mouth activity group

As indicated previously, traditional survey questions were often not answered as expected. However, while none of the traditional survey questions were 100% accurate in separating behavioral groups, expected patterns did emerge. Since both Chewers and Cruncher have a similar mouth action (using their teeth to break foods), it was not surprising that they were more similar to each other, in how they answered mouth behavior questions, than they were to Suckers or Smooshers. A similar pattern was seen with Suckers and Smooshers (i.e., answering more questions similarly).

There were some questions where a particular mouth behavior group answered the question significantly differently than all the other groups. There were other questions where one mouth behavior group rated questions differently than two of the other groups. Examples of questions that were different from at least two of the other three groups are shown in Table4. These results further validate the existence of mouth behavior groups.

**Table 4:** Response patterns of behavior groups shown from survey questions (*N* = 500)^1^^,^^2^^,^^3^

Chewers	Crunchers	Suckers	Smooshers
Prefer products they can chew^1^	Prefer hard crunchy cookies over soft chewy^1^	Prefer hard candy over soft^1^	Let cereal get soft or eat soft cereals like oatmeal^1^
Prefer chewy candy over hard candy^2^	Prefer hard granola bars over soft^1^	Like chocolate hard enough to suck on^1^	Prefer soft creamy candies over hard candy^1^
Would choose dried fruit that is chewy^2^	Eat ice cream right out of the freezer^1^	Like to suck a long time on candy^1^	Prefer thick creamy snacks over crispy^1^
Like chocolate with good chewing texture^2^	Like apples that are crisp^2^	Always have hard candy around^1^	Prefer flavored ice cream with no pieces^1^
Prefer cereals like Cheerios or flakes^2^	Like raw vegetables^2^	Like mints with some burn^1^	Chewing gum hurts their jaw^1^
Do not prefer chocolates hard enough to suck on^2^	Prefer ice cream with crunchy pieces^1^	Like high carbonation in drinks^1^	Like food that is soft and spreads through the mouth^1^
	Do not like to play with food in the mouth^1^		Smoosh foods that they could chew^2^

1 Significantly different (*P* < 0.1) than all other groups.

2 Significantly different (*P* < 0.1) from two of the three other groups.

3 This table was previously published in a book chapter (Jeltema et al. 2014).

While there were fewer questions for Chewers that were significantly different from all other groups, there were many questions that were different from one or two of the groups and many questions that differentiated Crunchers from Chewers. For example, Chewers were more likely than Crunchers to say:

- I like chewy cookies, let cereal soften, let ice cream soften, prefer chewy pieces in ice cream, choose soft chewy snacks, pick soft dried fruit
- I don't like granola, do like hard dried fruit, you can't hear me crunch as I eat

Similarly, there were other questions that differentiated Chewers from Suckers and Smooshers.

While many of these questions were able to show significant differences between groups, there were no questions where 100% of a mouth behavior group agreed. The highest agreement was in the 80's, (e.g., 89% of Crunchers indicated that they would rather have a crisp or crunchy snack over a creamy snack). Other questions, while still showing significant differences, might be in the 30's (Crunchers preferring granola cereals over flakes or soft cereals). This again illustrates the difficulty in using survey questions to type individuals who use complex mouth behaviors. These survey questions do, however, lend credence to the hypothesis that individuals with different mouth behaviors do make different product choices.

### Mouth behavior and product choice

Consumer packaged goods (CPG) companies know that “one size does not fit all”, and therefore they have a wide variety of line extensions for any given product, some based on flavor and others on texture. Confection is one area where there are many texture options. However, the reasons behind the existence of these segments of consumers had not been investigated. The following results illustrate how consumers make choices in a product to optimize the texture that best matches their mouth behavior.

Chocolate is an example of a product that is eaten differently. Some individuals chew chocolate, while others tend to hold it in their mouths and let it melt. As you would expect, these eating styles reflected mouth behavior group differences. There were also significant differences in how these groups responded to a series of questions on chocolate.

- Suckers—like chocolate that is hard enough to suck on— like to alternate chewing and sucking on chocolates
- Smooshers— like chocolate that melts fast
- Crunchers—like chocolate that contains nuts
- Chewers—like chocolate that has a good chewing texture

This illustrates how consumers consistently choose food options based on their mouth behavior. Understanding, not only how consumers eat foods differently but also how they are making these choices, is crucial to product development.

## Discussion and Implications

This research has shown that people differ in the ways that they like to manipulate food in their mouths and that this behavior drives texture preference and food choice. It should not be interpreted from these results that this implies that people would only choose to eat foods that easily allow a certain mouth behavior. It is more an indication of foods that are more often chosen (when there is a choice) or are more *delightful* or *satisfying*.

Individuals are believed to adapt the foods they are eating. For example, if a Cruncher is eating a soft food such as chocolate or a soft granola bar, they will more often choose a product with nuts or chips. A Chewer who is eating cereal will often add chewy ingredients like raisins or allow the cereal to soften slightly before eating it.

While there are foods that need to be chewed, such as meats and vegetables, for example, it is hypothesized that a person brings their mouth behavior preference to the food being eaten. For example, a Cruncher will more forcibly chew meat than will a Chewer. They will probably choose different meats or prefer the same meat cooked to different internal temperatures. A Sucker may prefer riper stone fruits that can be sucked on to release the juice before needing to be chewed. Interestingly, Suckers indicated that they were often dissatisfied with foods that they ate. Thirty percent said “I don't usually buy the foods that I prefer, it's more about what my family will eat and I just deal with it”. This could reflect the fact that there are fewer foods available that truly *delight* Suckers.

While people were found to have one predominant mouth behavior, they sometimes will choose an alternate behavior with certain foods. For example, a Cruncher may also occasionally enjoy smooshing, and have some foods that they sometimes (or partially) smoosh (like chocolates or ice cream). Only mouth behaviors that are *rejected* will reflect themselves in foods that are never or rarely chosen (if that food requires the use of that mouth behavior). For example, those individuals who reject smooshing will rarely eat semiliquid foods that cannot be chewed.

### Mouth behavior and oral processing

Research in oral processing has also shown that individuals can be grouped by how they process food (Brown and Braxton (2000); Engelen and van Doorn (in Engelen and de Wijk 2012). The relationship between groups found in oral processing and these mouth behavior groups needs to be studied. Future work will be needed to relate physical measurements of oral processing to these mouth behavior groups. Areas of interest should include, but not be limited to, mouth and jaw shape and size, salivary flow or salivary amylase, oral health, as well as fundamental mechanical or rheological properties of the foods.

### Influence of mouth behavior on desired sensation levels

It has been pointed out that individuals achieve the highest food sensations by using complex mouth actions (tongue, teeth, palate, etc.), and that this is done to maximize pleasantness (de Wijk et al. 2003). However, it has also been suggested that individuals balance chewing efficiency and perception in their choice of oral processing style (Engelen and de Wijk 2012).

Then how do individuals, like Suckers and Smooshers maximize their food sensations? Do different groups seek out products that are different in food sensation levels or do different groups want different levels of sensation?

While there is minimal data available at this time to answer these questions, the survey data suggest that individuals may seek (or avoid) different levels of sensation. The data suggest that Suckers are more likely to enjoy high levels of carbonation in their drinks and to enjoy mints with burn. This may indicate that these individuals use the increased trigeminal response (burning sensation) to help boost sensations. On the other hand, (maybe because they hold food in their mouths longer) both Suckers and Smooshers were more likely to indicate a dislike of hot food or cold drinks.

### Other potential mouth behavior groups

Not explored here were two other groups that were noted in the qualitative research. The first is a small group who seemed to want food to be in their mouths only a very short time. Given the option they would rather drink their food or take a pill. Interestingly, Smooshers were more likely to agree with this idea than any other group. The second group is a Fiddling group. Individuals may be Fiddlers in addition to their primary mouth behavior. Fiddlers can be Body Fiddlers (e.g., they always are tapping feet or tapping or clicking pens) or Mouth Fiddlers (e.g., they spend a lot of time playing with food in their mouths or they enjoy twirling or chewing on straws). Mouth Fiddlers probably exist in some, but not all mouth behavior groups (although least likely in Smooshers). Suckers who spend the most time with food in their mouths were most likely to like to play with food in their mouths.

### Implications for marketing and product design

The implications for marketing messages and on products designed to match that message are important. Product textures and messages can no longer be developed that will resonate with everyone. Since the different mouth behavior groups eat products differently, their ideas of what provides a good texture also differ. Not only will the groups desire different overall textures, that is creamy versus crunchy, but their definition of the textural words themselves will also differ. What is a good *crunch* or a good *chew* to one group will not be the same for another group.

Designers and developers need to understand these groups and use that knowledge to develop appropriate products for each of these mouth behavior groups. Many food companies are recognizing the importance of texture and are advertising products based on textural attributes such as crunchy, crispy, creamy, and so on. Testing for these products is done with the belief that attributes such as crunchy are not only meaningful, but also have a reliably consistent definition. The belief is (1) that most individuals like products that are, for example, crunchy and (2) that all individuals have the same understanding of what to expect of products that are called crunchy. This research suggests that neither of these beliefs is true. The qualitative findings suggest that what makes a good crunch for a Cruncher is very different than what makes a good crunch for a Chewer. Additionally, the advertising by some companies’ suggests that individuals seek different food textures depending on their emotional state, for example, when they are depressed or stressed. The findings suggest that this is in fact not the case, in that mouth behavior is inherent to the individual and does not depend on a person's emotional state. Additionally, products that allow multiple mouth behaviors will be more universally liked, while those that do not allow multiple behaviors, may only attract a certain type of person. No one food will be optimum for all mouth behaviors.

### Implications for the elderly

Qualitatively, those older individuals who have experienced dental issues indicated that they could no longer enjoy many of the foods that were preferred when they were younger. For example, Crunchers were not able to choose hard crunchy foods and were therefore forced to eat softer foods. While older persons have been identified as showing lower pleasure when eating because of their reduced ability to taste food, inability to use the preferred mouth behavior is hypothesized to also produce a reduced interest in food. Research conducted with elderly people with dentures showed that palatal coverage interferes with oral perception (Kremer et al. 2007). Those individuals with dentures rated food as less creamy. The reduced perception of texture was thought to be due to impairment of chewing and mouth movements. However, Kremer also found that compensatory changes in flavor or texture did not improve pleasantness. The current research indicates that, in the normal course of eating, individuals do exhibit unconscious behaviors to improve the texture of foods. This area of inquiry needs further study.

### Mouth behavior and obesity

Another area of inquiry is related to current issues in developed countries around obesity. Rolls et al. (2006) created a model that has been found to be very good at assisting in weight loss (the volumetric diet) as has the Weight Watchers approach to foods (Haupt 2014). It can be hypothesized that once an individual understands their specific mouth behavior requirements, they may be able to enhance their compliance with diet regimes and may actually be able to avoid certain weight gain scenarios (eating foods that are low in calories but not satisfying because they do not allow the right mouth behavior). By eating foods that most readily allow the preferred mouth behavior, individuals will be more satisfied and will be able to eat less.

### Mouth behavior and genetics

While there is currently no data to support this, it can be hypothesized that, while mouth behavior may correlate with gender and personality, it is more basic, and is something a person is born with (i.e., genetic). This hypothesis is supported by evidence that food cravings (Beckley and Moskowitz 2002; Moskowitz et al. 2002), taste sensitivity, and chewing behavior (Michon et al. 2009) differ by gender.

## Conclusion

The mouth behavior model may be related to chewing behaviors measured analytically. That suggests that it will be shown that an individual's mouth behavior allows that person to achieve their desired level of sensory stimulation. The creation of the JBMB™ Classification Tool as a reliable device for understanding individual mouth behavior provides a basis for other researchers to explore these ideas and others for a better understanding of human behavior and food interactions.
